# Complete Genome Sequence of Aeromonas caviae Strain MS6064, a *mcr-3*-Carrying Clinical Isolate from Japan

**DOI:** 10.1128/MRA.01037-20

**Published:** 2021-03-04

**Authors:** Liansheng Yu, Hiroki Kitagawa, Shizuo Kayama, Junzo Hisatsune, Hiroki Ohge, Motoyuki Sugai

**Affiliations:** a Antimicrobial Resistance Research Center, National Institute of Infectious Diseases, Tokyo, Japan; b Department of Antimicrobial Resistance, Graduate School of Biomedical and Health Sciences, Hiroshima University, Hiroshima, Japan; c Project Research Center for Nosocomial Infectious Diseases, Hiroshima University, Hiroshima, Japan; d Department of Surgery, Graduate School of Biomedical and Health Sciences, Hiroshima University, Hiroshima, Japan; e Department of Infectious Diseases, Hiroshima University Hospital, Hiroshima, Japan; University of Maryland School of Medicine

## Abstract

We report the complete genome sequence of *mcr-3*-carrying Aeromonas caviae strain MS6064, isolated from a blood sample from a Japanese patient. The strain carried *mcr-3* variant 3.38 and was borderline resistant to colistin (2 μg/ml).

## ANNOUNCEMENT

Aeromonas caviae is the most frequently isolated causative pathogen of *Aeromonas* bacteremia (1, 2). A. caviae strain MS6064 was isolated in 2014 from a blood sample from a 60-year-old Japanese woman diagnosed with polycystic kidney disease (2).

The strain, which had been kept in a glycerol (20%) stock at −80°C, was grown overnight on a Luria-Bertani (LB) agar plate, and a single colony was picked and transferred into LB broth at 37°C. Genomic DNA was extracted from the pellet using the Qiagen Genomic-tip 20/G kit (Qiagen). For Illumina sequencing, a library was constructed using the QIAseq FX DNA library kit (Qiagen), and paired-end sequencing (2 × 300 bp) was performed using the MiSeq reagent kit v3 on the MiSeq platform. A total of 1,274,512 paired-end reads were obtained from the MiSeq run after adaptor trimming. Trimming of low-quality reads and assembly (average coverage of 36×) were performed with Shovill v1.0.9 (https://github.com/tseemann/shovill). Long-read library preparation for MinION (Oxford Nanopore Technologies [ONT]) sequencing was performed using the SQK-RBK004 rapid barcoding kit (ONT) without DNA size selection, and sequencing was performed with MinKNOW software using a FLO-MIN106 R9.4 flow cell (ONT). Fast5 read files were base called and demultiplexed with Guppy v2.3.1 (ONT). Hybrid assembly of Illumina short reads and MinION long reads was performed using the hybrid assembler Unicycler v0.4.7 (3) with default parameters. The Unicycler pipeline automatically identified and trimmed overlaps for circular genomes and rotated the genome to begin with the *dnaA* gene. Sequence annotation and analysis were performed using PATRIC (https://www.patricbrc.org), ResFinder v3.2, and MLST v2.0 software (http://www.genomicepidemiology.org). BLASTn was used for the alignment of MCR-3 variants. Default parameters were used for all software unless otherwise specified.

The complete genome sequence of MS6064 contained a circular 4,578,485-bp chromosome, with a GC content of 61.29%. A total of 4,306 protein-coding genes, including 31 rRNAs and 121 tRNAs, were predicted by PATRIC. Genomic analysis showed that MS6064 was in sequence type 368 and contained various antibiotic resistance genes, including *aadA1*, *bla*_MOX-4_, *mcr-3*, *cat*, *sul1*, and *tet*(C). This novel *mcr-3* variant was designated *mcr-3.38*.

**FIG 1:**
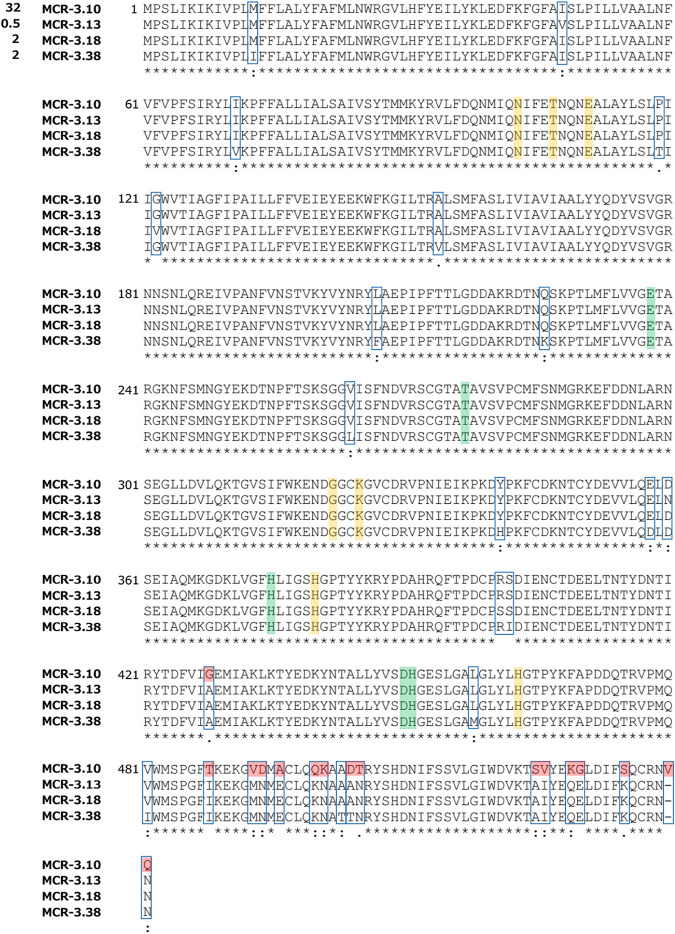
Protein sequence alignment of the novel MCR-3.38, compared with MCR-3.10, MCR-3.13, and MCR-3.18. The five Zn^2+^-interacting residues (E238, T277, H375, D450, and H451) and the seven possible phosphatidylethanolamine substrate-binding residues (N103, T107, E111, G322, K325, H380, and H463) are highlighted in green and yellow, respectively. The variant residues are indicated in blue boxes, and common variant residues among MCR-3.13, MCR3.18, and MCR3.38 are highlighted in red. Colistin MIC values are indicated to the left of each sequence.

Some *mcr-3*-carrying isolates are susceptible or borderline resistant to colistin, but there was no clear relationship between colistin MICs and the phylogeny of MCR-3 variants. Among the four available A. caviae *mcr-3*-positive strains, only the *mcr-3.10*-positive strain showed a high colistin MIC (32 μg/ml) (4); all of the other isolates, which were *mcr-3.13*, *mcr-3.18* (5), and *mcr-3.38* positive, had a low MIC (0.5 or 2 μg/ml) (Fig. 1). Thirty-three amino acid substitutions in MCR-3.10 are present in at least one of MCR-3.13, MCR3.18, and MCR3.38, but none of them corresponded to potential zinc-interacting residues or possible phosphatidylethanolamine substrate-binding residues (6). Common variations of MCR-3.13, MCR3.18, and MCR3.38, compared with MCR-3.10, are clustered in the C-terminal region. The molecular mechanism of borderline colistin resistance of A. caviae carrying *mcr-3.38* remains unknown.

### Data availability.

The sequence data for A. caviae MS6064 have been deposited in the DDBJ Sequence Read Archive (DRA) under accession number DRA010575. The nucleotide sequence of *mcr-3.38* has been deposited in GenBank under accession number MT787344.
